# Short-Term Forecasting of Daily Confirmed COVID-19 Cases in Malaysia Using RF-SSA Model

**DOI:** 10.3389/fpubh.2021.604093

**Published:** 2021-06-14

**Authors:** Shazlyn Milleana Shaharudin, Shuhaida Ismail, Noor Artika Hassan, Mou Leong Tan, Nurul Ainina Filza Sulaiman

**Affiliations:** ^1^Department of Mathematics, Faculty of Science and Mathematics, Universiti Pendidikan Sultan Idris, Tanjung Malim, Malaysia; ^2^Data Analytics, Sciences & Modelling (DASM), Department of Mathematics & Statistics, Faculty of Applied Sciences and Technology, Universiti Tun Hussein Onn Malaysia, Parit Raja, Malaysia; ^3^Department of Community Medicine, Kulliyyah of Medicine, International Islamic University Malaysia, Kuantan, Malaysia; ^4^Geoinformatic Unit, Geography Section, School of Humanities, Universiti Sains Malaysia, Gelugor, Malaysia

**Keywords:** COVID-19, eigentriples, forecasting, recurrent forecasting, singular spectrum analysis, trend, window length

## Abstract

Novel coronavirus (COVID-19) was discovered in Wuhan, China in December 2019, and has affected millions of lives worldwide. On 29th April 2020, Malaysia reported more than 5,000 COVID-19 cases; the second highest in the Southeast Asian region after Singapore. Recently, a forecasting model was developed to measure and predict COVID-19 cases in Malaysia on daily basis for the next 10 days using previously-confirmed cases. A Recurrent Forecasting-Singular Spectrum Analysis (RF-SSA) is proposed by establishing L and *ET* parameters via several tests. The advantage of using this forecasting model is it would discriminate noise in a time series trend and produce significant forecasting results. The RF-SSA model assessment was based on the official COVID-19 data released by the World Health Organization (WHO) to predict daily confirmed cases between 30th April and 31st May, 2020. These results revealed that parameter *L* = 5 (T/20) for the RF-SSA model was indeed suitable for short-time series outbreak data, while the appropriate number of eigentriples was integral as it influenced the forecasting results. Evidently, the RF-SSA had over-forecasted the cases by 0.36%. This signifies the competence of RF-SSA in predicting the impending number of COVID-19 cases. Nonetheless, an enhanced RF-SSA algorithm should be developed for higher effectivity of capturing any extreme data changes.

## Introduction

In 2020, Malaysia has witnessed the outbreak of a virus called Severe Acute Respiratory Syndrome Coronavirus 2 (SARS-CoV-2) or COVID-19 that is highly infectious to human's respiratory system, hepatic system, gastrointestinal system, and neurological disorders. This virus can spread between humans, livestock, and wild animals, such as birds, bats, and mice (1, 2). Belonging to the coronavirus family, this novel virus type is accountable as a cause for mild to moderate colds. The SARS-CoV-2 may cause severe acute respiratory illnesses that result in fatality for various cases. The symptoms of COVID-19 are cough, fever, nose congestion, shortness of breath, and occasionally, diarrhea (3). In Malaysia, the virus started to spread swiftly by the end of January 2020. Since then, the Crisis Preparedness Response Centre (CPRC) of Malaysia's Ministry of Health (MOH) has begun recording and reporting the cases. The COVID-19 statistics is updated based on the total active cases, recoveries, and casualties attained daily from the MOH website.

The worst scenario of SARS-CoV-2 infection to individuals is fatality. Nevertheless, information on the mechanism of the spread of the virus or how it affects a patient seems to be in scarcity. The Centres for Disease Control and Prevention (CDC) has verified the COVID-19 human-to-human transmission on 30th January 2020. As noted by the CDC, COVID-19 can spread via droplet, close contact with infected patients, and contact with surfaces or objects that has the particles of the virus. It has been stipulated that 2–14 days or longer as the incubation period of COVID-19 with 5 days on average (4).

As the impact of this virus is severe, therefore it is important to be able to detect the pattern and forecast the spread of confirmed cases is very crucial. For an instance, Zhao et al. (5) had proposed a mathematical model to approximate the actual COVID-19 cases, including those unreported, for the first half of January 2020. It was deduced that the unreported cases count was 469 between 1st and 15th January 2020. Next, the estimation of cases from 17th January 2020 onwards revealed that the case numbers astonishingly encountered a 21-fold upsurge. This epidemic was predicted to reach its peak in late February and subside by late April based on the SEIR model combined with a machine-learning artificial intelligence (AI) method (6). Subsequently, Tang et al. (7) prescribed a mathematical model that could estimate the risk of COVID-19 transmission. Based on this, the potential number of the basic reproduction was 6.47. It also forecasted the total of 7 day confirmed cases with 23rd−29th January 2020 time interval. Consequently, the estimated peak was after 2 weeks from the initial date of 23rd January 2020.

In order to estimate the prolonged COVID-19 human-to-human transmission, data obtained from 47 patients were analyzed and resulted in a transmission rate of 0.4 (8). If the duration between the symptom detection and the patient hospitalization was halved from the tested study data, the transmission rate could reduce to 0.012. In another study, an estimation of SIR model was exhibited for the COVID-19 outbreak in Malaysia to predict the short-term daily COVID-19 cases (9). The study reported a transmission rate of 0.22 by considering that an infected individual can spread the virus to another individual within 4 days. This human-to-human transmission rate of 4 days should be highly considered, or even viewed as conservative.

Furthermore, various researchers have employed Box-Jenkins time series analysis model in predicting future cases of COVID-19 (10–12). For an instance, Rauf and Hannah (12) found out ARIMA (2, 2, 2) model produced the most accurate results compare to others for cases in India. Meanwhile, Jibrin et al. (11) recommended that the Autoregressive Fractional Integral Moving Average (ARFIMA) model should be used for further analysis of daily COVID-19 new cases. Rauf and Hannah (12) found an upward trend of the spread of COVID-19 in Nigeria based on ARIMA (1,1,0) model and more. According to Jianxi (13), the developed predictive model of COVID-19 cases must be considered on several factors such as intertwined human, social, and political factors. Due to that, predictive monitoring paradigm was proposed, which synthesized the prediction and monitoring of the daily COVID-19 cases in the study area. Another forecasting method to predict COVID-19 cases is based on machine learning approaches (14–17). Jianxi (13) stated that the hybridization model of machine learning approaches produces better performances in predicting cumulative COVID-19 cases with high daily incidence. In addition, the climatic variables were employed as inputs for proposed forecasting machine learning models.

Most of the previous studies focuses on the forecasting of future cases COVID-19. However, the analysis of this pandemic pattern is equally important. The proposed method suggested by Yogesh (18) considered the trend of new cases of COVID-19 in developing forecasting model. Nevertheless, this model didn't ensure that the trend and noise components in the data were clearly separated before the forecasting values were generated. The suitable analytical tools to assess the global change pattern with uncertainty metrics seem to be rather limited and seldom applied systematically, as it is often presented as an operational pattern worldwide. Systematically tracking and observing the infectious disease in a specific population and presented chronologically at high temporal resolution can lead to a modern and sophisticated methodology to perform in-depth data analysis. Hence, suitable analytical methods for time series data may be used if cases of health outcomes are assembled and aggregated with time units (e.g., weekly or daily basis).

Singular Spectrum Analysis (SSA) is a superb and effective alternative to address trend components, substantially minimize noise, and unravel the temporal structure of data minus preliminary manipulation (19). Generally, SSA represents univariate time series transformed into eigenvectors and eigenvalues of any trajectory matrix. The SSA refers to a multidimensional analog of principal component analysis (PCA), which is transformed into time series. One function of the SSA is to separate the time series data into noise, trend, and seasonal categories by decomposing the time series eigen, and later, reconstructing them into group selection (20).

The SSA, essentially, transforms a single dimension time series into trajectories with multiple dimensions via PCA [Singular Value Decomposition (SVD)], as well as reconstruction (approximation) of chosen Principal Components. However, the separation of the components in this approach depends on the parameters, which is the selection of window length, *L*, to form trajectory matrix and identifying the number of leading eigentriples (*ET*), based on eigenvector plot (21). This separation is crucial in this model to ensure that the trend, seasonal, and noise components are easily separated.

Although SSA lacks parametric description and highly relies on the length of time series, these flexible SSA models can recreate the asymmetric shapes of a trend, hence allowing better prediction of seasonal peaks than can harmonic models. This model, when compared to others, is easy to use, dismisses specification of models of time series and trend, enables extraction of trend in the presence of noise and oscillations, and involves only two parameters to determine the accuracy and flexibility in predicting outcomes (22).

As the SSA models are seldom used to assess epidemiological data, this study is set to introduce the SSA model based on combining forecasting elements of time series analysis known as Recurrent Forecasting-Singular Spectrum Analysis (RF-SSA). To ensure that this developed model produces significant forecasting results, the selection of the parameter for this model, which are the window length, *L* and the amount of leading eigentriples used, *ET*, was identified using several tests. The SSA was used in this study as a base approach to build the forecasting model. The next sections describe the data in detail, followed by several sections that present the methodology, the results and discussion, and finally, the conclusion.

## Data

Daily COVID-19 prevalence data from 25th January to 29th April 2020 were gathered from MOH records. As this COVID-19 is a newly-founded virus; no COVID-19 data was available from the previous year. The suspected COVID-19 cases were diagnosed by using the Reverse Transcription Polymerase Chain Reaction (RT-PCR) technique and were confirmed as COVID-19 case-counts. All fully anonymized, laboratory-confirmed cases were abstracted on COVID-19, in which 5,945 cases represented COVID-19 infections in all 13 states and 3 federal territories in Malaysia, as recorded by MOH.

**Figure 1:**
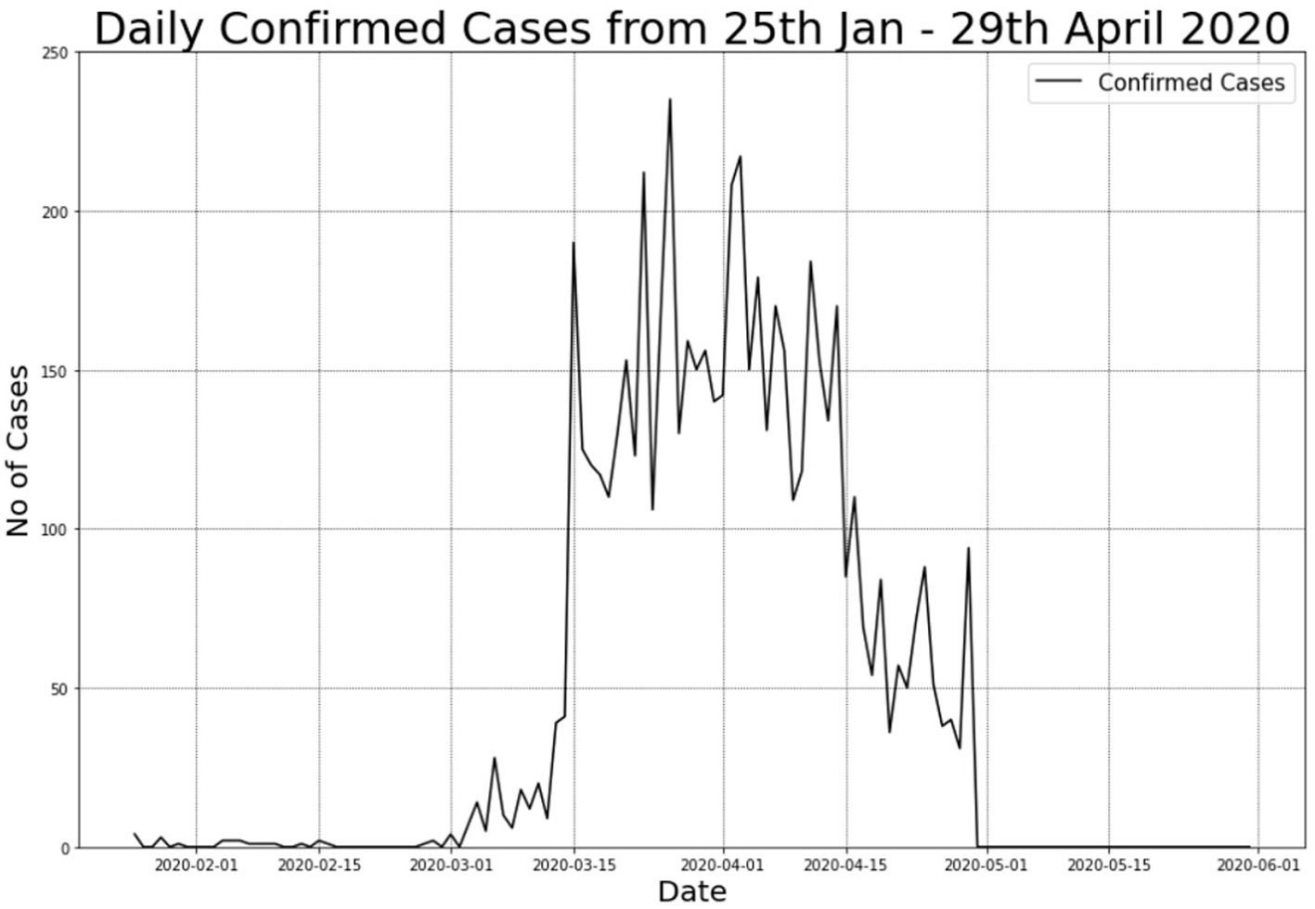
COVID-19 daily confirmed cases in Malaysia from 25th January to 29th April 2020.

Figure 1 illustrates the total positive cases for COVID-19. The figure displays a significant spike in the number of positive cases that resulted in the 2nd wave of COVID-19 pandemic in Malaysia. With this substantial number, the Malaysian Government had announced a Movement Control Order (MCO) that took place from 18th to 31st March 2020. The MCO was later extended to the 4th phase.

**Figure 2:**
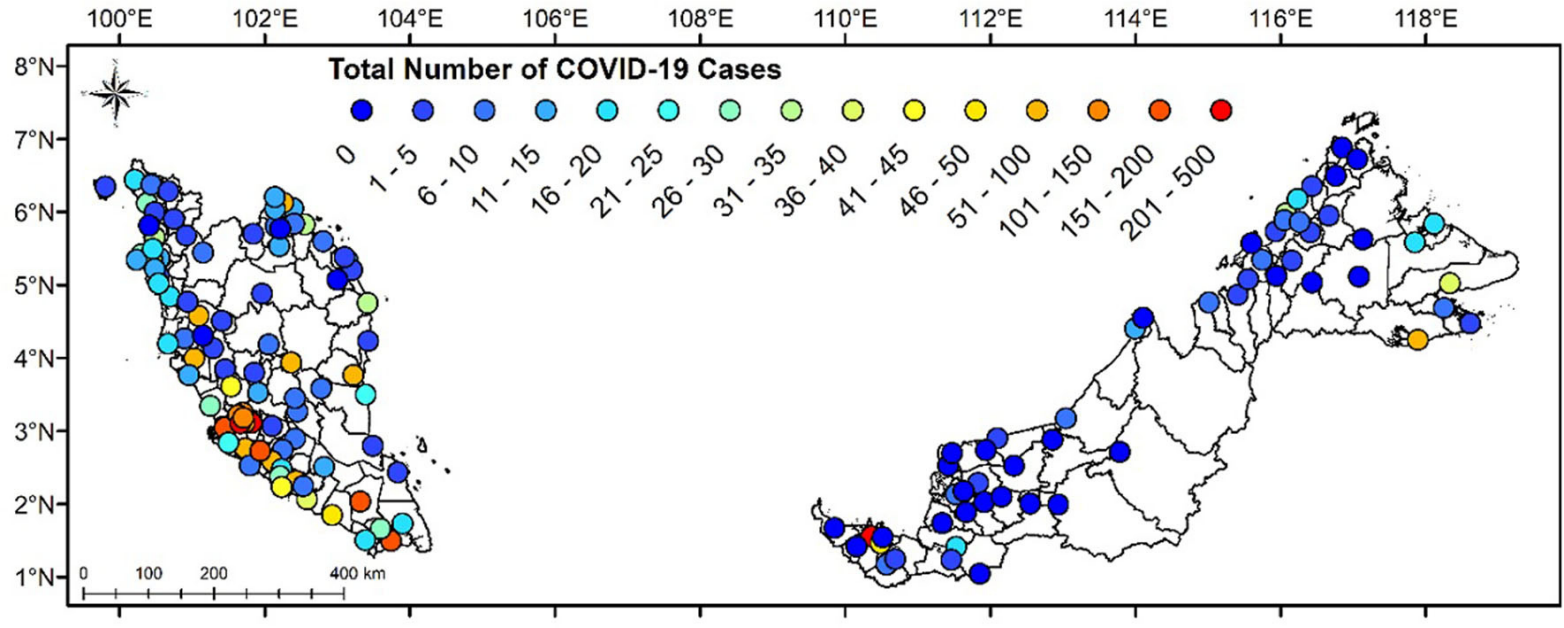
State classification based on number of COVID-19 cases in Malaysia from 25th January to 29th April 2020.

Figure 2 portrays the observed number of cases for COVID-19 for the last 96 days in Malaysia. The MOH had categorized four zones of COVID-19 areas in Malaysia based on the areal cases number. According to the National Security Council (MKN), the four zones are: (i) green zone for areas with no positive case, (ii) yellow zone for areas with one to 20 positive cases, (iii) orange zone for areas with 21 to 40 positive cases, and (iv) red zone for areas with more than 40 positive cases (23).

The projection and estimate daily cases of COVID-19 obtained were impacted by the definition of the case reported to CPRC daily, whereby a large number of pending result test daily was definitely influential to a non-consistent increase in the number of confirmed cases. The increased prediction cases are supported by several of the biggest clusters identified by the MOH, such as Seri Petaling Tabligh Cluster, Wedding Kenduri in Bandar Baru Bangi, Seri Petaling Sub-Cluster in Rembau, Italy Cluster in Kuching, and Church Fellowship Cluster in Sarawak. The new confirmed cases were extremely spiking as the target of biology samples were taken directly from highly susceptible infected population.

## Materials and Methods

This section elaborates on the specifics of SSA model and its components.

### Singular Spectrum Analysis (SSA) Model

The SSA is a model-free approach that can be applied to all types of data, regardless of Gaussian or non-Gaussian, linear or non-linear, and stationary or non-stationary (24). The daily COVID-19 data can be decomposed into several additive components via SSA, which could be defined in the forms of trend, seasonal, and noise components (25). The possible application areas of SSA are diverse (26–28). The SSA is composed of two complementary stages, known as the stages of decomposition and reconstruction (29).

### Stage 1: Decomposition

The two steps in the decomposition stage are embedding and SVD. This stage decomposes the series to obtain eigen time series data.

***Step I: Embedding*. **The first step in basic SSA algorithm is embedding, which refers to constructing the original time series into a sequence of lagged vector of size window length, *L* by forming lagged vectors, *K* = *T* − *L* + 1 of size *L*. $X_{i}={\left(x_{i},\ldots ,x_{i+L-1}\right)}^{T}$ (1 ≤ *i* ≤ *K*).
  The trajectory matrix of the series 𝕏 is
  (1) $$\begin{array}{l} X=\left(X_{1},\ldots ,X_{K}\right)\;={\left(x_{ij}\right)}_{i,j=1}^{L,K}=\left(\begin{array}{cccc} x_{1} & x_{2} & x_{3} & \cdots \;x_{K}\\ x_{2} & x_{3} & x_{4} & \cdots \;x_{K+1}\\ x_{3} & x_{4} & x_{5} & \ldots \;x_{K+2}\\ \vdots & \vdots & \vdots & \ddots \;\vdots \\ x_{L} & x_{L+1} & x_{L+2} & \cdots \;x_{T} \end{array}\right)\text{​​​​​} \end{array}$$
  The rows and columns of **X** are subseries of the original one-dimensional time series data and lagged vectors *X*_*i*_ are the columns of the trajectory matrix **X**.
***Step II: Singular Value Decomposition (SVD)*. **In the second step, the trajectory matrix in Step I is decomposed to obtain the eigen time series based on their singular values using SVD. The following represents the SVD of the trajectory matrix, *X*_*i*_ where λ_1_, …, λ_*L*_ are denoted as the eigenvalues of *XX*^*T*^ where singular values are arranged in a descending order such that (σ_1_ ≥ σ_2_ ≥ ⋯ ≥ σ_*L*_) and by *U*_1_, …, *U*_*L*_the corresponding eigenvectors. The SVD of *X* can be represented as *X* = *X*_1_ + ⋯ + *X*_*L*_, where $X_{i}=\sqrt{\lambda_{i}}U_{i}V_{i}^{T}$ and $V_{i}=\frac{X^{T}U_{i}}{\sqrt{\lambda_{i}}}$ if (λ_*i*_ = 0 we set *X*_*i*_ = 0). The set of $\left(\sqrt{\lambda_{i}},U_{i},V_{i}\right)$ is called the *i* − *th* eigentriple (*ET*) of the matrix *X*_*i*_, and $\sqrt{\lambda_{i}}$ are the singular values of the matrix *X*_*i*_.

### Stage 2: Reconstruction

Grouping and diagonal averaging are the two steps in the reconstruction phase. Here, the original series are reconstructed for further analysis, including forecasting.

***Step 1: Grouping*. **Here, the trajectory matrix is divided into dual groups—trend, seasonal and noise components. Upon setting *I* = {*i*_1_, …, *i*_*p*_} be a group indices, *i*_1_, …, *i*_*p*_ where (*p* < *L*). Then the matrix **X**_*I*_ corresponding to the group I is defined **X**_*I*_ = **X**_*i*1_ + … + **X**_*ip*_. The indices set {1, …, *L*} is divided into *m* disjoint subsets; *I*_1_, …, *I*_*m*_, based on the division of elementary matrices into groups of *m*. The retrieved matrices are calculated for *I* = *I*_1_, …, *I*_*m*_ which called is eigentriple grouping corresponding to the representation of **X** = **X**_*I*1_ + … + **X**_*Im*_.
***Step 2: Diagonal averaging*. **The last step in SSA refers to the transformation of each matrix in the grouped decomposition into new series of length, *T*.

- Let **Z** be *L* × *K* matrix with *z*_*ij*_, 1 ≤ *i* ≤ *L*elements, 1 ≤ *j* ≤ *K*. Set L^*^ = min(*L, K*), K^*^ = max(*L, K*), and *N* = *L* + *K* − 1. Let z_ij_^*^ = *z_ji_* if *L* < *K* and z_ij_^*^ = *z_ji_* otherwise. With diagonal averaging, matrix **Z** is transferred into *z*_1_, …, *z*_*T*_ based on the following formula:
  (2) $$\begin{array}{l} z_{k}\{\begin{array}{c} \frac{1}{k}\sum_{m=1}^{k}z_{m,k-m+1}^{*}\;1\leq k<L^{*}\\ \frac{1}{L^{*}}\sum_{m=1}^{L^{*}}z_{m,k-m+1}^{*}\;L^{*}\leq k\leq K^{*}\;\\ \frac{1}{T-k+1}\sum_{m=k-k^{*}+1}^{T-K^{*}+1}z_{m,\;k-m+1}^{*}\;K^{*}<k\leq N \end{array} \end{array}$$
- Upon applying the diagonal averaging in equation above to the resultant matrix, **X**_*Ik*_, reconstructed series of ${\tilde{Y}}_{T}^{\left(k\right)}=({\tilde{y}}_{1}^{\left(k\right)},\ldots ,{\tilde{y}}_{T}^{\left(k\right)}$ is produced. The initial series of 𝕐_*T*_ = {*y*_1_, *y*_2_, …, *y*_*T*_} is decomposed into the total of *m* reconstructed series, $y_{t}=\overset{m}{\underset{k=1}{\sum }}{\tilde{y}}_{t}^{\left(k\right)}$. The reconstructed series generated by elementary grouping refers to ‘elementary reconstructed series.

### Stage 3: Forecasting

To perform SSA forecasting, the time series should satisfy the linear recurrent formula (LRF). Time series *Y*_*T*_ = (*y*_1_, …, *y*_*T*_) satisfies LRF of order d if:
  (3) $$\begin{array}{l} y_{t}=a_{1}y_{t-1}+a_{2}y_{t-2}+\ldots +a_{d}y_{t-d},\;t=d+1,\ldots ,T \end{array}$$
  In this study, Recurrent SSA (RSSA) was used for forecasting purpose because it is a popular approach to predict data (30, 31). The algorithms described below are detailed in Golyandina et al. (32).
  Let us assume that $U_{j}^{\nabla }$ is the vector of the first *L* − 1 components of eigenvector *U*_*j*_, while π_*j*_ is the last component of *U*_*j*_(*j* = 1, …, *r*). Denoting $v^{2}=\overset{r}{\underset{j-1}{\sum }}\pi_{j}^{2}$, coefficient vector ℜ is defined as follows:
  (4) $$\begin{array}{l} ℜ=\frac{1}{1-v^{2}}\overset{r}{\underset{j=1}{\sum }}\pi_{j}\;U_{j}^{\nabla } \end{array}$$
  Upon considering the prior notation, the forecast of RSSA (ŷ_*T*+1_, …, ŷ_*T*+*M*_) can be attained by
  (5) $$\begin{array}{l} {\hat{y}}_{i}=\{\begin{array}{c} {\tilde{y}}_{i},\;i=1,\ldots ,T\\ {ℜ}^{T}Z_{i},\;i=T+1,\ldots ,T+M \end{array} \end{array}$$
  where, $Z_{i}={\left[{ŷ}_{i-L+1},\ldots ,{ŷ}_{i-1}\right]}^{T}$ and ỹ_1_, …, ỹ_*T*_, are the values of reconstructed time series (noise reduced series).

### SSA Parameter Selection

Extraction of trend from the original time series data relies on the window length, *L*, to form the trajectory matrix in SSA. Improper values selection for parameter *L* may yield unfinished reconstruction, which may potentially mislead the forecasting results. It has been stipulated that *L* should be large enough, but not greater than half of the number of observations understudy at $\frac{T}{2}$ (33). The appropriate window length selection depends on the structure of time series data and the current problems (34). Generally, there is no guide to determine the proper *L* in a dataset. The separability conditions for shorter time series may be restrictive due to the SVD properties used in estimating the signal component in SSA. Therefore, in this study, several *L* namely $\frac{T}{2},\frac{T}{5},\frac{T}{10},\frac{T}{20}$, were investigated on COVID-19 data based on performance error, which refers to Root Mean Square Error (RMSE).

Another parameter to be considered when using the SSA approach is the amount of leading *ET*by inspecting the eigenvector plot. This plot is the eigenvector of the SVD of trajectory matrix for time series data. The one-dimensional graphs of eigenvectors were inspected to identify the trend components. The trend has a complex form when both the trend and noise components were not properly distinguished. It is highly possible that lack of separability caused the mix-up between the components. This information may serve as a guideline to identify proper grouping for component separation of the trend and noise appropriately. This reflects a link between the stages of decomposition and reconstruction.

### Evaluating Separabality in Time Series Data

A key concept when studying SSA is separability, which signifies how the varied components of time series may be differentiated from each other to enable further analysis. When working with SSA method in numerous study fields, separability becomes a vital mean (35). The separability impact can result in appropriate decomposition and component extraction. The *w-correlation* technique measures the separability between two distinct components of the reconstructed time series.

The *w-correlation* reflects the weighted correlation among components of reconstructed time series that offers highly useful information to both separate and identify groups for the reconstructed components (36). The elements of the time series terms are indicated by the weights into trajectory matrix. This ranges between 0 and 1, whereby components that are well-separated slant toward 0, whereas slant toward 1 for otherwise. The *w-correlation* matrix looks into grouped decomposition among the reconstructed components. The matrix formulation of *w-correlation* is as follows:

(6) $$\begin{array}{l} \rho_{12}^{w}=\frac{\langle X^{\left(1\right)},X^{\left(2\right)}\rangle w}{∥X^{\left(1\right)}∥w∥X^{\left(2\right)}∥w} \end{array}$$

where $∥X^{\left(i\right)}∥w=\sqrt{\langle X^{\left(i\right)},X^{\left(i\right)}\rangle w},\;i=1,2,\;\langle X^{\left(1\right)},X^{\left(2\right)}\rangle w=\overset{N-1}{\underset{i=0}{\sum }}w_{i}x_{i}^{\left(1\right)}w_{i}^{\left(2\right)}$, and weights *w*_*i*_ are defined below:

Let L^*^ = min(*L, K*) and K^*^ = max(*L, K*). As a result,

(7) $$\begin{array}{l} w_{i}=\{\begin{array}{c} i+1\;for\;0\leq i\leq L^{*}-1,\\ L^{*}\;for\;L^{*}\leq i\leq K^{*},\;\\ T-i\;for\;K^{*}\leq i\leq T-1. \end{array} \end{array}$$

The graphic illustration of *w-correlation* is composed of white-black scale, whereby white represents correlation that is small, whereas black denotes correlation between the series components near to value 1.

### Evaluation Performances

**Figure 3:**
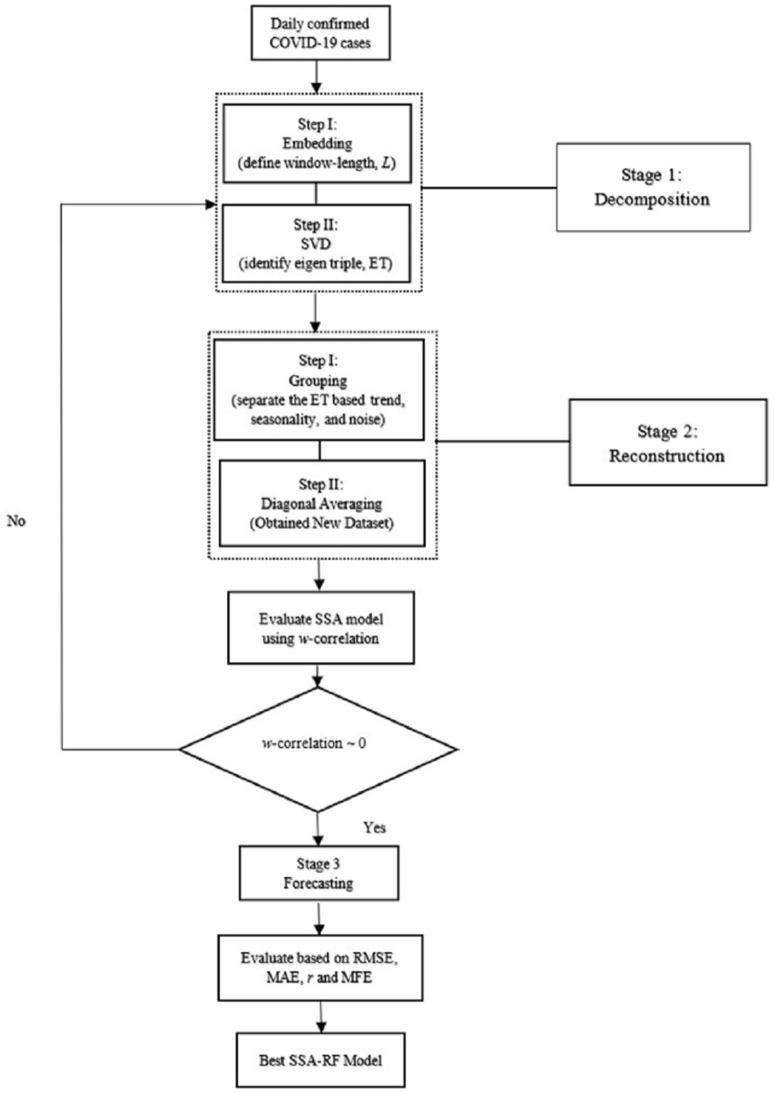
Flow chart of developed forecasting, model of RF-SSA.

In this study, four types of evaluation performances are applied to evaluate the accuracy of the predicted output for the forecasting models. The measurements used in this study are Mean Absolute Error (MAE), Mean Forecast Error (MFE), and Root Mean Square Error (RMSE), whereby, the best model is selected based on the smallest values for that measurements. Meanwhile, the Pearson Correlation Coefficient (*r)* value is based on a range from +1 to −1. A value of *r* that close to +1 or −1 indicated that the two observed variables are related to each other. Concurrently, a value of 0 indicates that there is no association between two observed variables. The equations for each of the evaluation performances are shown as follows:

(8) $$\begin{array}{l} MAE=n^{-1}\left[\overset{n}{\underset{i=1}{\sum }}\left|y_{t}-\hat{y}\right|\right] \end{array}$$

(9) $$\begin{array}{l} MFE=n^{-1}\left[\overset{n}{\underset{i=1}{\sum }}\left(y_{t}-\hat{y}\right)\right] \end{array}$$

(10) $$\begin{array}{l} RMSE=n^{-2}{\left[\overset{n}{\underset{i=1}{\sum }}{\left(y_{t}-\hat{y}\right)}^{2}\right]}^{-0.5} \end{array}$$

(11) $$\begin{array}{l} r=\frac{n\left(\sum_{i=1}^{n}x_{t}y_{t}\right)-\left(\sum_{i=1}^{n}x_{t}\right)\left(\sum_{i=1}^{n}y_{t}\right)}{\sqrt{\left[n\left(\sum_{i=1}^{n}{x_{t}}^{2}\right)-{\left(\sum_{i=1}^{n}x_{t}\right)}^{2}\right]\left[n\left(\sum_{i=1}^{n}{y_{t}}^{2}\right)-{\left(\sum_{i=1}^{n}y_{t}\right)}^{2}\right]}} \end{array}$$

where *y*_*t*_ is the actual values at time *t*; *y*_*t*_ is the predicted values at time *t*; *n* is the number of observations. Flow chart of developed forecasting model based on SSA as shown in Figure 3.

## Results and Discussion

### Decomposition and Reconstruction

In the initial stage of this study, COVID-19 data were decomposed into components by using the SSA model, which required identification of (*L, ET*) parameter pair. Here, *L* denotes the compromise between statistical confidence and information. The suitable *L* value should resolve the varied oscillations embedded in the original signal.

The performance of the SSA results was determined by assessing the *w-correlation* at distinct window length, *L*. The *w-correlation* calculated the separability among noise, trend, and seasonal (components of reconstructed time series). Here, *L* = *T*/2, *T*/5, *T*/10, and $\frac{T}{20}$, which represent *L*= 48, 19, 10, and 5, respectively, for *T* based on 96 daily cases on COVID-19 data had been selected. The scales were selected to fit the data of the time series, apart from striking a balance to achieve a proper lag vector sequence.

**Figure 4:**
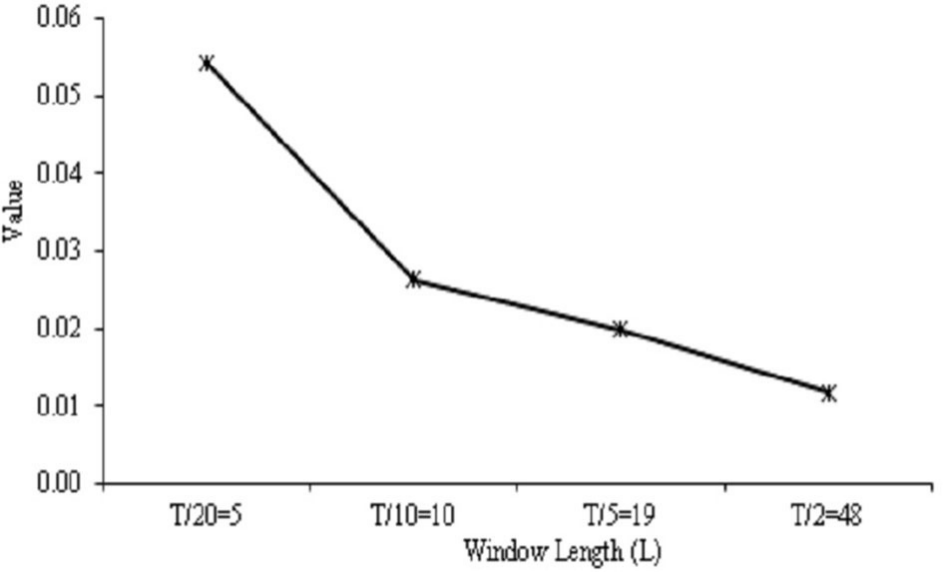
Effect of *w*-correlation based on SSA using COVID-19 data at varied window lengths.

In Figure 4, the *w*-*correlation* is presented based on SSA using daily cases of COVID-19 data at varying window lengths. The *w*-*correlation* displayed a declining trend when the total window length declined for SSA approach. The correlations among trend and other components should be close to zero for extraction of trend. This means; the distinct window lengths have an impact on the component's separability. Besides, the SSA was directed to the lowest *w-correlation* at *L* = *T*/20; signifying the best separability among the reconstructed components as it was the closest to zero.

**Figure 5:**
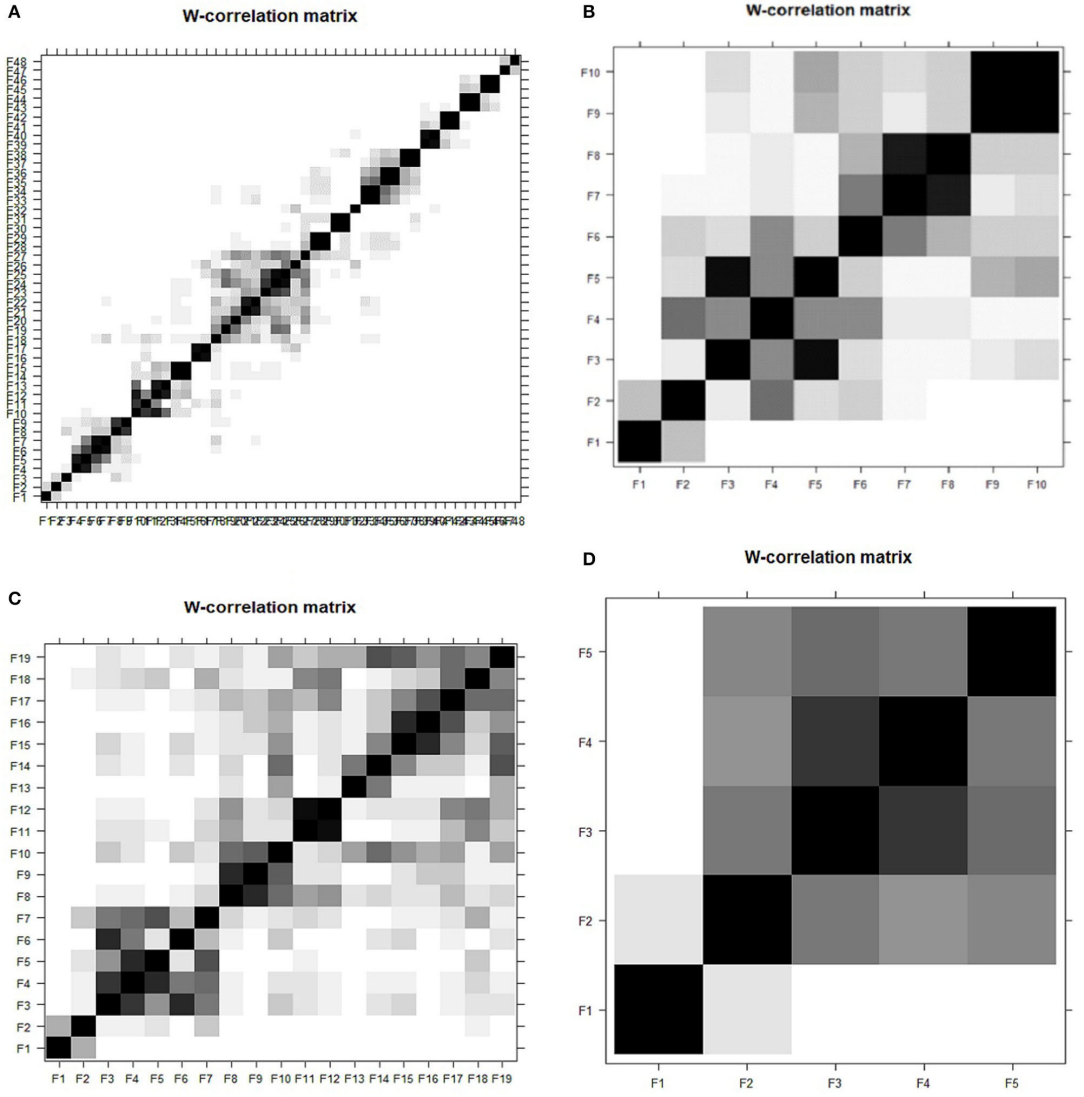
**(A–D)** *w-correlation* plot using SSA with varied windows length **(A)** *L* = 48 **(B)** *L* = 19 **(C)** *L* = 10 **(D)** *L* = 5.

The graphs in Figures 5A–D illustrate the heat-plot of different window lengths, *L*, based on *w-correlations* using the SSA approach. The heat-plot of *w-correlation* for the reconstructed components based on white-black scale ranges between 0 and 1 (37). Huge correlation values among the reconstructed components exhibited the possibility of the components to form a group while corresponding to the same component. As illustrated in Figure 5, the shade of each square represents the *w-correlation* strength between two components. Meanwhile, Figures 5A–C portrays the tendency of the components to form correlation with other components despite signifying weak correlation. Subsequently, this denotes that the components of trends are still, to some extent, mixed with the noise and seasonal components in SSA and it was rectified by the small window length, *L* = 5, which is evidently demonstrated in Figure 5D for better separability.

Table 1 presents the reconstructed time series components varied window length. The lowest RMSE was observed from $L=\frac{T}{20}$, which had the smallest value amongst other *L*, indicating its suitability based on short-time series of the outbreak data. Meanwhile, the high RMSE values were reported in this study due to the high model variance for small sample set.

**Table 1:** Comparison of Singular Spectrum Analysis Prediction Performance for Several Window Length (*L*).

**Window Length, L**	**RMSE**
*T*/2 = 48	29.51
*T*/5 = 19	29.67
*T*/10 = 10	23.97
*T*/20 = 5	19.12

**Figure 6:**
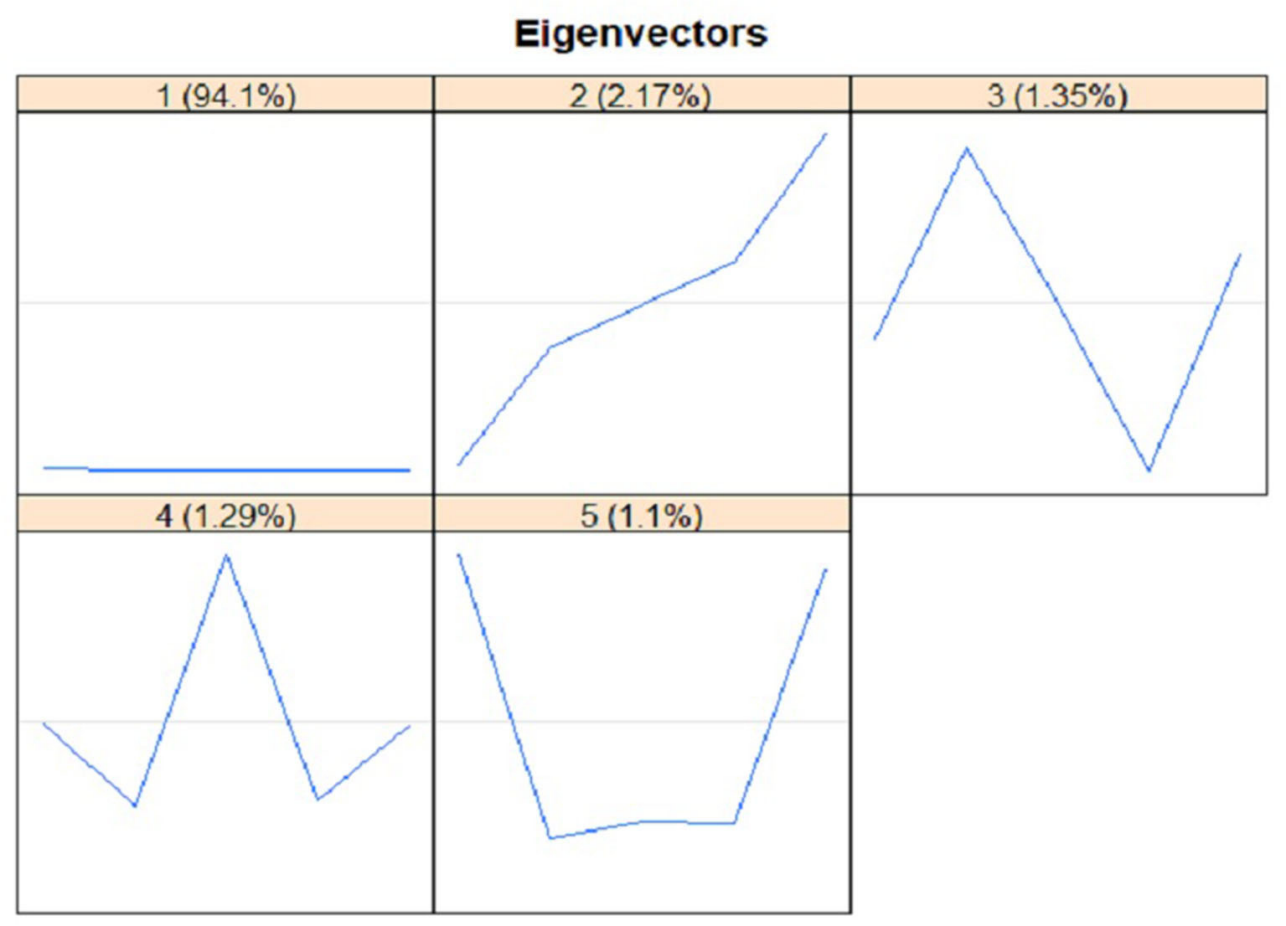
Eigenvectors Plot using Singular Spectrum Analysis.

**Figure 7:**
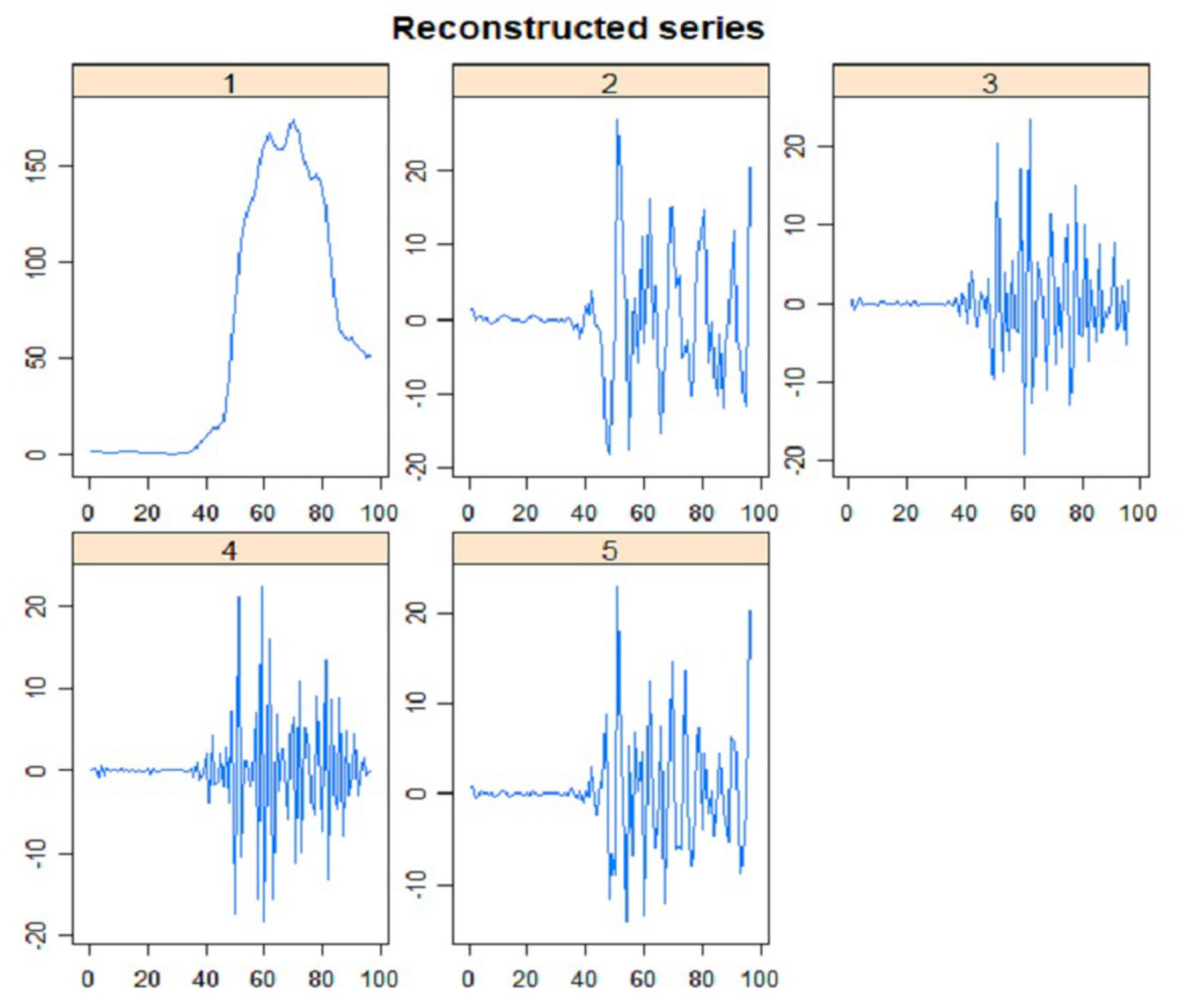
First stage: Elementary Reconstructed Series (L = 5).

The plot of five main eigenvectors is displayed in Figure 6. Such plot is beneficial to choose an appropriate group for the components of time series data, especially to separate the components of noise, trend, and seasonal. The retrieved information may be further analyzed in the step of grouping in RF-SSA. The component of trend was identified from eigenvector plot, in which seasonal and trend components have sine waves indicated by the slow cycles found in the graph (high frequency). Meanwhile, the component of noise was represented by the saw-tooth found in the graph (low frequency). The leading eigenvector has nearly continual coordinates, thus corresponding to a pure smoothing by Bartlett filter (38, 39). The reconstruction result by each of the five *ET* is presented in Figure 7. The two figures verified the compatibility of the first and second *ET* with the trend, whereas the remaining *ET* had the noise component, thus irrelevant to trend.

**Figure 8:**
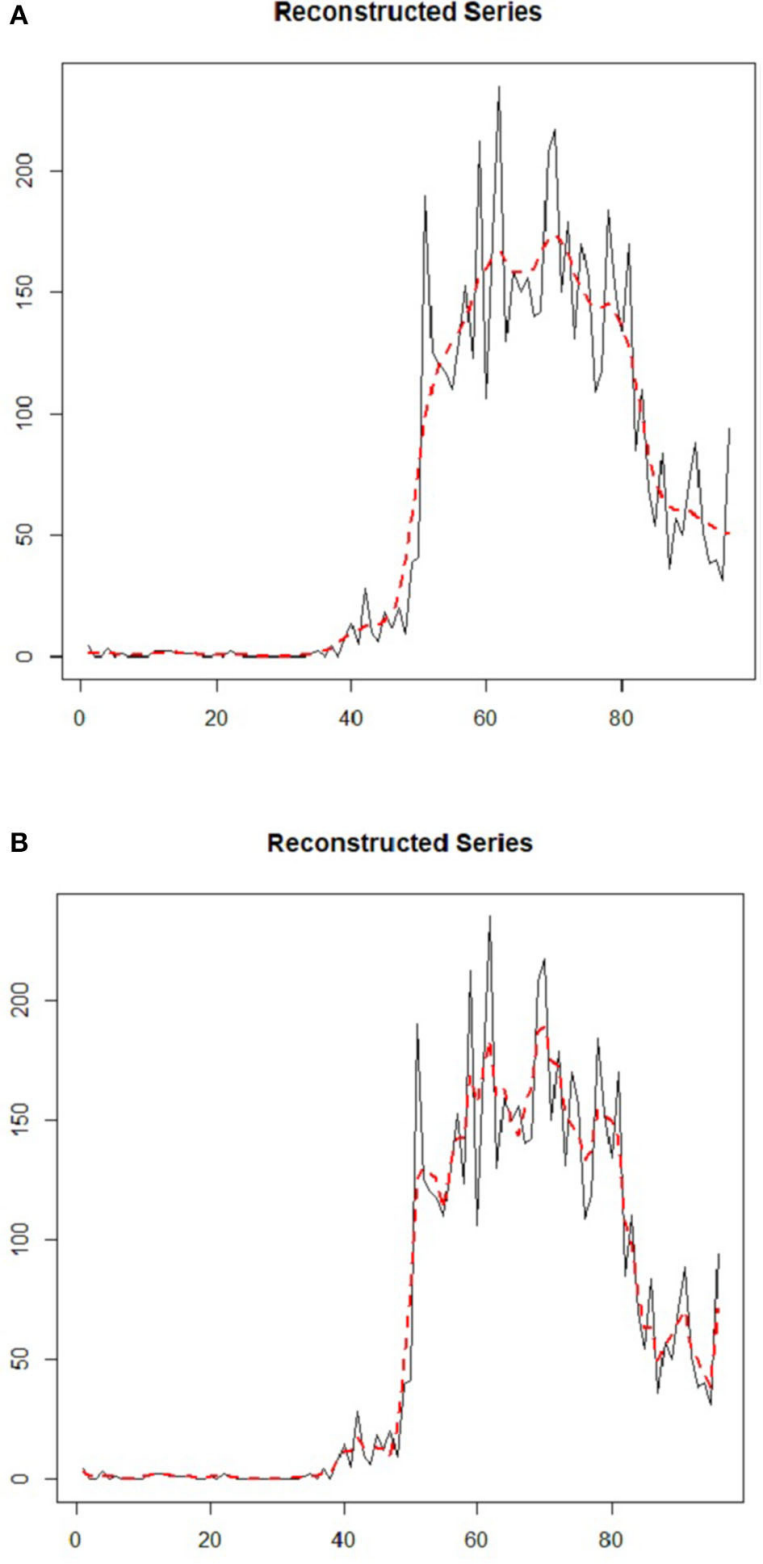
**(A,B)** Daily COVID-19 cases of reconstructed components from extracted trends using SSA at **(A)** L = 5, ET 1 **(B)** *L* = *5*, ET 2.

Figure 8 demonstrates the components of the reconstructed time series plot from the trend extracted via RF-SSA for daily COVID-19 cases in Malaysia. The reconstructed series is the new dataset derived from the original data, which is clear from noise. It is a crucial aspect in SSA to ensure that the forecasting results are precise and accurate (40). The component of trend in the time series data was used to observe the occurrence of the cases trend and pattern, as it was randomly-tabulated as per daily cases (see Figure 8). In Figures 8A, 7, the trend was precisely generated by a leading *ET*, which coincided with the initial reconstructed component exhibited in Figure 8. The trend in Figure 8B was precisely generated by both leading *ET*, which coincided with the first and second reconstructed components shown in Figure 8. The dashed and straight lines on the plot denote the reconstructed series based on the extracted trend component from SSA and the COVID-19 original time series data, respectively. The plot of reconstructed time series components, produced by both leading *ET*, abides by the original COVID-19 data although noise component was omitted for *L* = 5 for daily COVID-19 cases in Malaysia.

**Figure 9:**
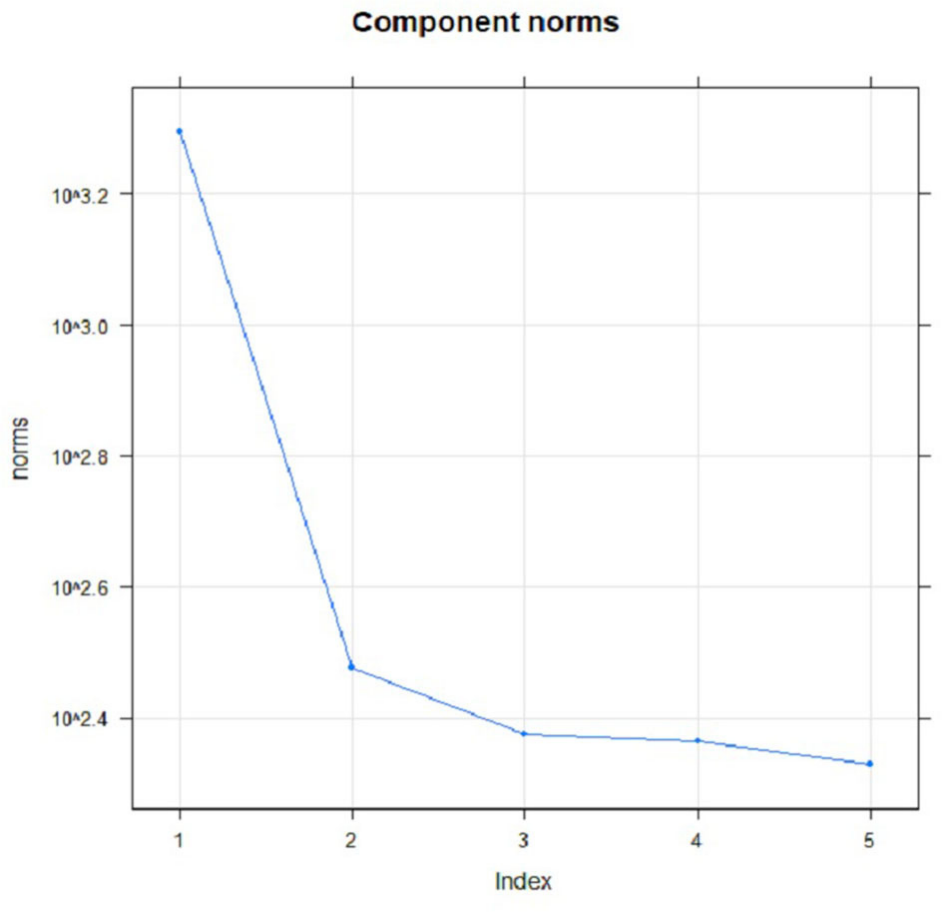
Logarithms of five eigenvalues.

**Figure 10:**
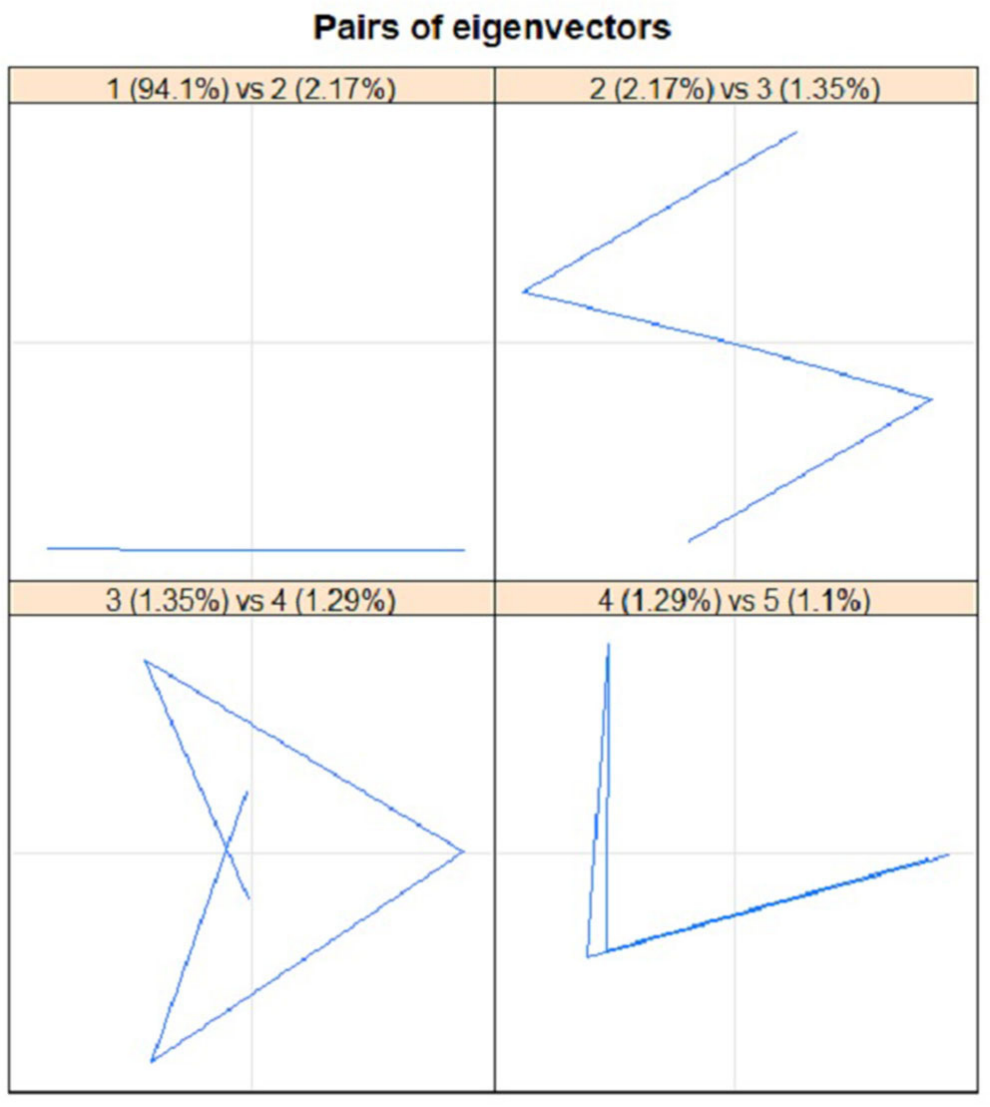
Plots of eigenvectors (EV) pairs: 1-EV and 2-EV, 2-EV and 3-EV, 3-EV and 4-EV, as well as 4-EV and 5-EV for COVID-19 cases.

For proper identification of seasonal series components, the graph of eigenvalues and scatterplots of eigenvectors were applied. In order to determine the seasonal series components using eigenvalues plot, several steps were produced by approximately equal eigenvalues. Figure 9 portrays the plot of the logarithms of the five singular values for the COVID-19 cases in Malaysia. It clearly showed that no step produced by approximately equal eigenvalues that corresponded to a sine wave. The scatterplot of eigenvectors displays the regular polygons yielded by a pair of eigenvectors to demonstrate that the series components have produced seasonality components. Based on Figure 10, no pair of eigenvectors produced regular polygons. This confirmed that the COVID-19 data in Malaysia were not influenced by the seasonality since both figures did not have sine wave.

### Forecasting Daily COVID-19 Cases Using SSA-RF

As mentioned in the previous section, the daily COVID-19 cases in Malaysia were first decomposed and reconstructed using SSA model. The next step in this study is to predict the future cases of COVID-19 in Malaysia. In this stage, an SSA forecasting algorithm known as Recurrent Forecasting were used accordingly. From hereafter, the model are known as SSA-RF. Table 2 presents the summary statistics from the experiment analysis of SSA-RF at several windows length.

**Table 2:** SSA-RF Prediction Performance Several Window Length (*L*).

***L***	**MAE**	***r***	**MSE**	
*T*/2 = 48	19.3706	0.9086	−2.8249	Over-forecast
*T*/5 = 19	19.3706	0.9086	−2.8249	Over-forecast
*T*/10 = 10	14.8890	0.9402	0.0067	Under-forecast
*T*/20 = 5	11.2549	0.9619	0.1920	Under-forecast

**Figure 11:**
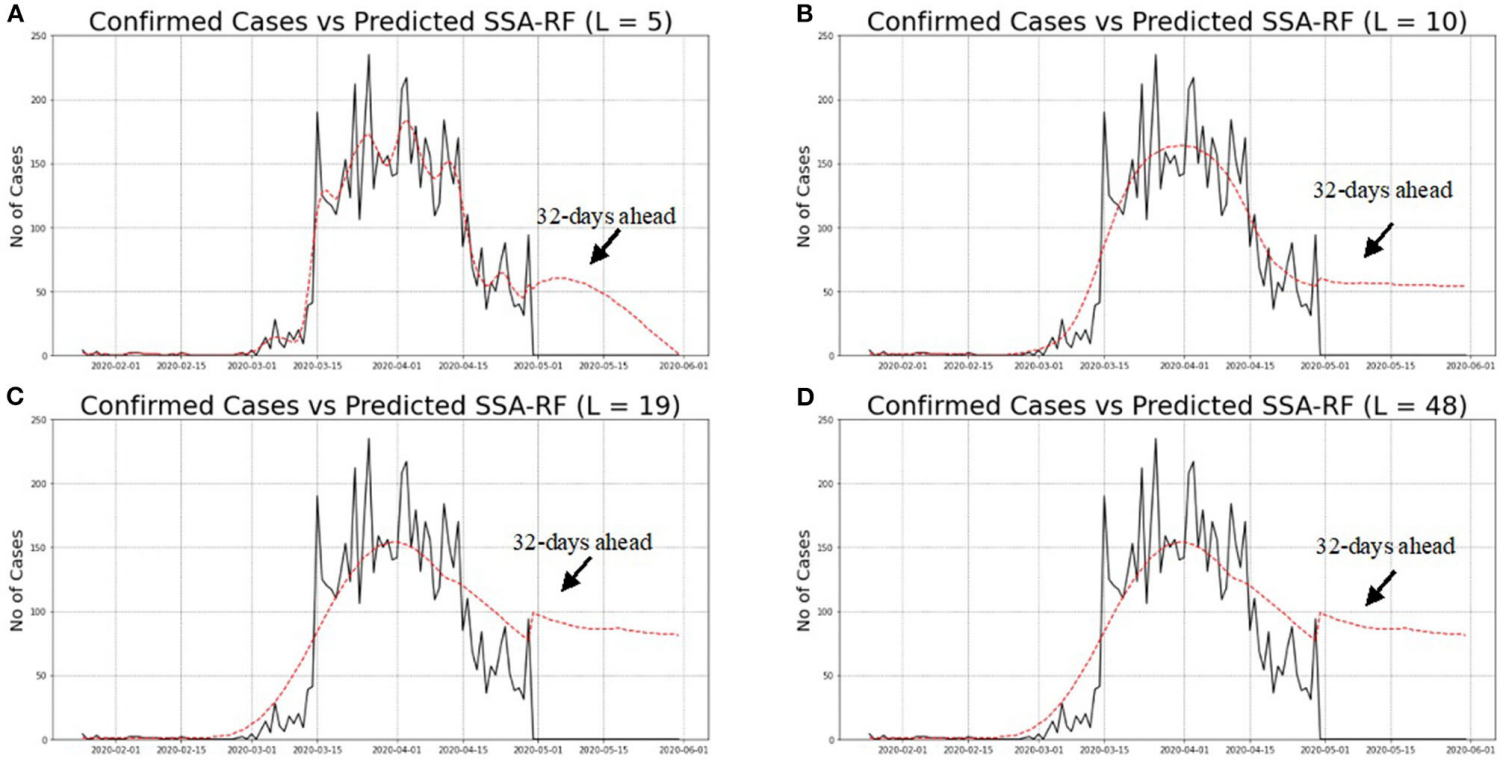
**(A–D)** Predicted SSA-RF and confirmed cases of COVID-19 in Malaysia for Various Windows Length *(L)*.

Looking at Table 2, it is apparent that the best performances can be obtained from *L* = 5 that has the lowest MAE of 11.2549 with the highest *r* of 0.9619, indicating superb correlation between confirmed and predicted cases. Moreover, the MFE shows that the SSA-RF algorithm with L = 5, tends to under-forecast daily COVID-19 cases by 0.1920%. Meanwhile, the second-best model is observed from SSA-RF with L = 10 where RMSE is 23.9652, MAE of 14.8890, *r* of 0.9402 with MFE of 0.0067%. Meanwhile, *L* = 19 and *L* = 48 has the worst performances among all models whereby MAE and *r* for both models are 19.3706 and 0.9086, respectively. Furthermore, MFE statistical results showed that both models are over-forecast by 2.82%. Visual inspection on these models performances are presented in Figures 11A–D.

Based on Figures 11A–D, it is a clear indication that SSA-RF models able to capture general pattern of non-linear increasing trend of daily confirmed cases of COVID-19 in Malaysia. Detailed analysis from Figure 11A found out that model with *L* = 5 performed better that other models whereby the model able to follow the actual pattern of daily confirmed cases of COVID-19. Meanwhile, as can be seen from Figures 11B–D, other models which are *L* = 10, *L* = 19, and *L* = 48 unable to follow the actual pattern of the observed data. This is a clear indication that the models performed poorly as compared to *L* = 5 model.

Next, the SSA-RF models were used to predict future cases starting from 30th April to 31st May 2020. At the time of this study, the historical cases from 25th January to 29th April 2020 were used and the future 32 days ahead of COVID-19 cases had been predicted accordingly. Figures 11A–D illustrates the confirmed cases from 25th January to 29th April 2020 and the forecasted daily cases until 31st May 2020. It is worth noting that the figures display a noticeable but faint decreasing pattern from 5th April 2020 onwards. One of the contributing factors for the decreasing trend was due to the MCO announced by the Malaysian Government which took place on 18th March 2020. The above figures also illustrate the predicted values of 32 day ahead using SSA-RF algorithm against confirmed cases of COVID-19 in Malaysia. Despite the encouraging statistical finding based from the historical data and lower under-forecast value; the SSA-RF models failed to capture the sudden drop in the COVID-19 cases, which is considered to have never happened before. This sudden drop was highly likely due to the MCO that was extended to phase-4, which ended on 12th May 2020.

During the MCO, Malaysians were advised to stay at home as much as possible to minimize the spread of further COVID-19 infections. All schools and most workplaces were closed, and they were directed to work from home except for essential services. Traveling ban, restriction movement order including interstate movement, restriction on gatherings, and public transport closure were imposed strictly by the government. Active case detection was continued, followed by isolation of the cases, and the close contacts were tested and quarantined to further curb the spread of COVID-19. All these actions successfully plateaued and reduced the number of COVID-19 cases (Figures 11A–D). In addition, the cases were reduced due to the incubation period of the virus between 2 to 14 days, and the recent findings from WHO has stated that after 5–10 days of the infection, the infected individual starts to gradually produce neutralizing antibodies which will decrease the risk of transmission to others (41, 42). WHO has also reported three research that found the inability of SARS-CoV-2 virus to be cultured after 7–9 days of onset of symptoms (43, 44). From all the latest findings, WHO has concluded that after 14 days, the patients are not likely to be infectious (45). The government's decision to extend the MCO up to 12th May had successfully plateaued and reduced the curve as it provides sufficient time to break the virus transmission.

Furthermore, the figures showed that different window length suggested a different forecasted value of future cases. For an instance, SSA-RF with L = 48. Nineteen and 10 predicted that there will be insignificant changes in the number of future cases, while SSA-RF with L = 5 showed there will be a significant drop in the future cases. Other than that, the model also suggested that Malaysia will reach single digit in COVID-19 cases by early June 2020. However, the model unable to predict the date for total eradication of COVID-19 cases. This is consistent with WHO which indicated that this virus will not be eradicated even after the vaccine is found. It might persist to be endemic in certain countries and will need cooperation on a global scale and leveraging tools such as contact tracing and disease surveillance to defeat COVID-19.

### Limitation of SSA-RF Model

Some limitations of this study, which should be emphasized when using the SSA-RF model in assessing the pandemic data in Malaysia, are as follows:

- The SSA-RF model works best when the data exhibit a stable or consistent pattern over time with a minimum amount of outlier. This can help to obtain accurate and precise results for future predictive cases.
- The sudden spike in data leads to low performance of forecasting results using this predictive SSA-RF model.
- The SSA-RF model is mainly used to project future values using historical time series data for short-term forecast.
- Recurrent forecasting approach is a better contender than vector approach for forecasting both short and medium time series data of SSA. However, under such scenarios, it is advisable that users also evaluate the performance of forecasting SSA approach on their data to arrive at a complete picture.
- Although SSA able to capture the pattern of the Coronavirus COVID-19 cases, however, its ability in predicting the cases accurately is still need to be investigated further.
- Different observed behavior of a dataset might influence the selection of window length.
- This model did not take into account the effect of incubation period in transmission of the virus, the effect of the government measures to curb the spread of COVID-19.

## Conclusion

This study assessed the applicability of SSA-RF model in predicting the COVID-19 cases in Malaysia. The application of this model is specifically advantageous for the health authorities in terms of flattening the curve by devising prompt and effective strategies. This model allows the health authorities to comprehend the outbreak pattern better. The pattern retrieved from the SSA-RF model can be applied to forecast the outbreak cases growth pattern in Malaysia. The parameters used in this model were window length, L, and the total of *ET* employed for reconstruction, r. The results revealed that parameter L = 5 (*T*/20) was suitable for short time series outbreak data and the appropriate number of leading *ET* s to obtain was crucial as it affected the forecasting outcomes. Overall, the results showed that the SSA-RF model could forecast this pandemic with reasonable accuracy as the model had under-forecasted by 0.1920% with high correlation values between confirmed and predicted cases. Nevertheless, the SSA-RF model failed to capture the sudden drop in COVID-19 cases, likely due to the MCO that was extended to 12th May 2020. In order to improve the accuracy of the model, more information is required to better predict the COVID-19 cases for a long period. In the meantime, case definition and data collection must be maintained in real-time to enhance the RF-SSA for further evaluation. It is suggested that the SSA-RF model is enhanced to enable the model to capture sudden and rapid changes in the dataset.

## Data Availability Statement

The raw data supporting the conclusions of this article will be made available by the authors, without undue reservation.

## Author Contributions

SS and SI conceived the presented idea, developed the theory, and performed the computations. NH, MT, and NS verified the analytical methods and supervised the findings of this work. All authors discussed the results and contributed to the final manuscript.

## Conflict of Interest

The authors declare that the research was conducted in the absence of any commercial or financial relationships that could be construed as a potential conflict of interest.
